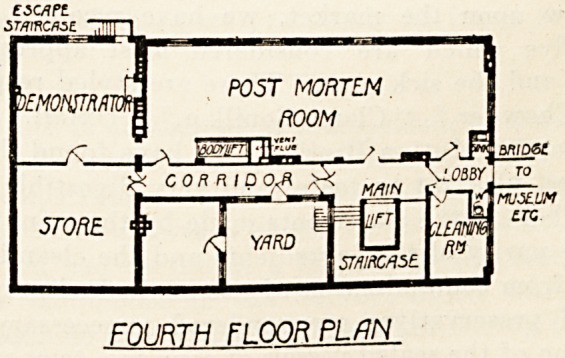# The New Pathological Block of St. Bartholomew's Hospital

**Published:** 1909-07-10

**Authors:** 


### HOSPITAL ADMINISTRATION.

### CONSTRUCTION AND ECONOMICS.

### THE NEW PATHOLOGICAL BLOCK OF ST. BARTHOLOMEW'S HOSPITAL.

We publish to-day a further instalment of our description of the important work of reconstruction which is in progress at this ancient institution.

The new building, which is devoted to the purposes of pathological teaching and research, faces West Smithfield and adjoins the Museum Library and Medical School buildings erected some years back. Including the basement there are six stories. The basement contains the mortuary chapel where the body of a deceased patient can be viewed by

GROUND FLOOR PLAN

E. B. I'ANSON M.A. F.R.I.B.A.

ARCHITECT

7a LAURENCE POUNTNEY HILL E.C.

relatives and friends or by a coroner's jury. Adjoining is a waiting-room. The mortuary proper is a large cold chamber capable of holding eighteen bodies, and provided with a refrigerating apparatus. There is a special lift by which bodies can be raised to the top story, which contains the post-mortem room.

On the ground floor are the school administration offices and a common room for the staff. The

FIRST FLOOR PLAN.

handsome furniture of these rooms has been provided by private subscription among the members of hospital and medical school staffs. The first floor contains a large laboratory for clinical pathology, two research laboratories, rooms for the pathologist and demonstrator and room for media with assistants' room adjoining. The second floor provides three large laboratories for pathological histology, bacteriology, and for junior demonstrators. On the third floor is the Kanthack Memorial pathological library, laboratory for chemical pathology, and lecturers' private room. On the fourth floor is the post-mortem room with rooms adjoining for demonstrators, storage, etc. The post-mortem room, which, as pointed out above, is connected with the

SECOND FLOOR PLAN

mortuary by a lift, contains six necropsy tables—four of slate and two of porcelain—and an adequate supply of washing-troughs and water-sprays.

The building is probably the most complete of its kind ever erected, and is striking evidence of the

THIRD FLOOR PLAN

increasing importance of the part played by the pathological department in the modern hospital. This aspect of the subject is further emphasised by the fact that altogether some 25 men will be con-

FOURTH FLOOR PLAN

stantly at work in pathological research, apart from the general students.

The building was designed and carried out under the superintendence of Mr. E. B. I'Anson, F.R.I.B.A., the architect to the hospital.

## Figures and Tables

**Figure f1:**
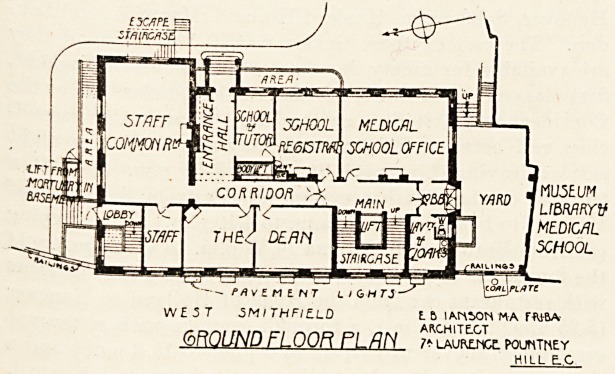


**Figure f2:**
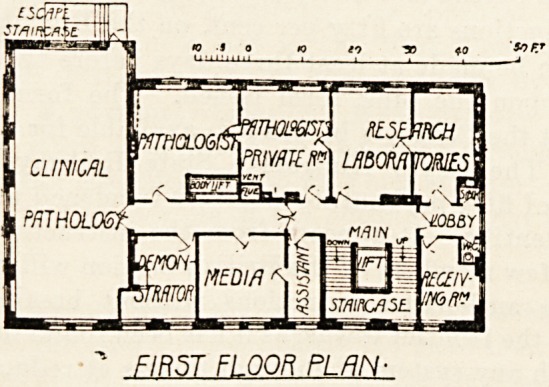


**Figure f3:**
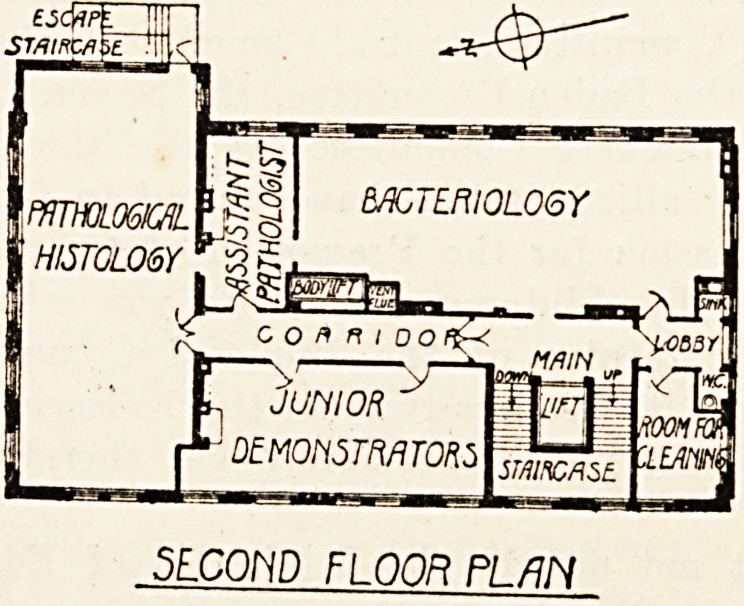


**Figure f4:**
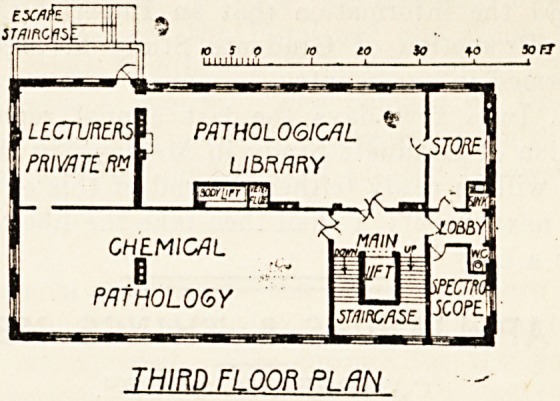


**Figure f5:**